# Nutritional status impacts dengue virus infection in mice

**DOI:** 10.1186/s12915-020-00828-x

**Published:** 2020-08-27

**Authors:** Christina Chuong, Tyler A. Bates, Shamima Akter, Stephen R. Werre, Tanya LeRoith, James Weger-Lucarelli

**Affiliations:** 1 Department of Biomedical Sciences and Pathobiology, VA-MD College of Veterinary Medicine, Virginia Tech, Blacksburg, VA USA; 2 Present Address: Department of Bioinformatics and Computational Biology, School of Systems Biology, George Mason University, Fairfax, VA USA; 3 Department of Population Health Sciences, VA-MD College of Veterinary Medicine, Virginia Tech, Blacksburg, VA USA

**Keywords:** Dengue virus, Arbovirus, Nutrition, Malnutrition, Nutritional immunology, Disease severity

## Abstract

**Background:**

Dengue virus (DENV) is estimated to infect 390 million people annually. However, few host factors that alter disease severity are known. Malnutrition, defined as both over- and undernutrition, is a growing problem worldwide and has long been linked to dengue disease severity by epidemiological and anecdotal observations. Accordingly, we sought to establish a mouse model to assess the impact of nutritional status on DENV disease severity.

**Results:**

Using transiently immunocompromised mice, we established a model of mild dengue disease with measurable viremia. We then applied it to study the effects of healthy weight, obese, and low-protein diets representing normal, over-, and undernutrition, respectively. Upon infection with DENV serotype 2, obese mice experienced more severe morbidity in the form of weight loss and thrombocytopenia compared to healthy weight groups. Additionally, obesity altered cytokine expression following DENV infection. Although low protein-fed mice did not lose significant weight after DENV2 infection, they also experienced a reduction in platelets as well as increased spleen pathology and viral titers.

**Conclusions:**

Our results indicate that obese or undernourished mice incur greater disease severity after DENV infection. These studies establish a role for nutritional status in DENV disease severity.

## Background

Worldwide, dengue virus (DENV) infects approximately 390 million people each year and is arguably the most significant arthropod-borne viral (arbovirus) threat [1]. Mild dengue disease symptoms include fever accompanied by myalgia, headache, and retro-orbital pain. Severe dengue disease manifestations include hemorrhage and shock, generally accompanied by thrombocytopenia, producing some 500,000 hospitalizations annually, mostly among children. Four DENV serotypes circulate worldwide, and prior infection with another serotype is the most significant risk factor for developing severe dengue disease [2–4]. DENV remains a neglected tropical disease despite its growing incidence globally; identifying additional risk factors for severe disease is essential for reducing DENV disease burden.

Alongside the increasing incidence of DENV, malnutrition—in the form of under- and overnutrition—is a growing concern globally. According to the World Health Organization (WHO), more than 1.9 billion adults are overweight or obese, while 462 million are underweight [5]. Several studies have found a link between DENV disease severity and nutritional status in humans. Undernutrition has mostly been associated with protection from severe dengue manifestations, such as dengue hemorrhagic fever (DHF) or dengue shock syndrome (DSS) [6–8]. Conversely, in several reports [9–11], obesity has been associated with increased disease severity following DENV infection in humans. Poor nutritional status alters host immunity, impairing an effective response to infection. In particular, obesity is a chronic inflammatory state that leads to decreased memory T and B cell production during influenza infection, resulting in impaired viral clearance and increased disease severity [12, 13]. Undernutrition, specifically low protein intake, referred to as protein-energy malnutrition or PEM throughout the rest of the manuscript, reduced influenza-specific cellular immunity and increased disease severity [14]. Despite growing evidence that malnutrition has a significant impact on disease severity for several pathogens, the epidemiological link between DENV disease severity and nutritional status has not been confirmed in empirical studies using mouse models.

To this end, we developed a transiently immunocompromised mouse model of mild DENV infection using wild-type mice treated with type I interferon (IFN) receptor (IFNAR) blocking antibody. Following infection with low-passage DENV1 or DENV2 strains, mice generated consistent viremia levels and mild hematological changes despite not showing overt clinical signs or weight loss. We then fed mice different diets to induce obesity or PEM before infection with DENV2 and observed increased disease severity in obese and PEM mice compared to healthy weight controls. Our results confirm the detrimental effects of poor nutritional status on disease severity in DENV infections and suggest that nutritional status should be considered in public health programs aimed at preventing severe dengue disease.

## Results

### Transiently blocking the type I IFN receptor renders mice susceptible to infection with DENV1 and DENV2

**Fig. 1:**
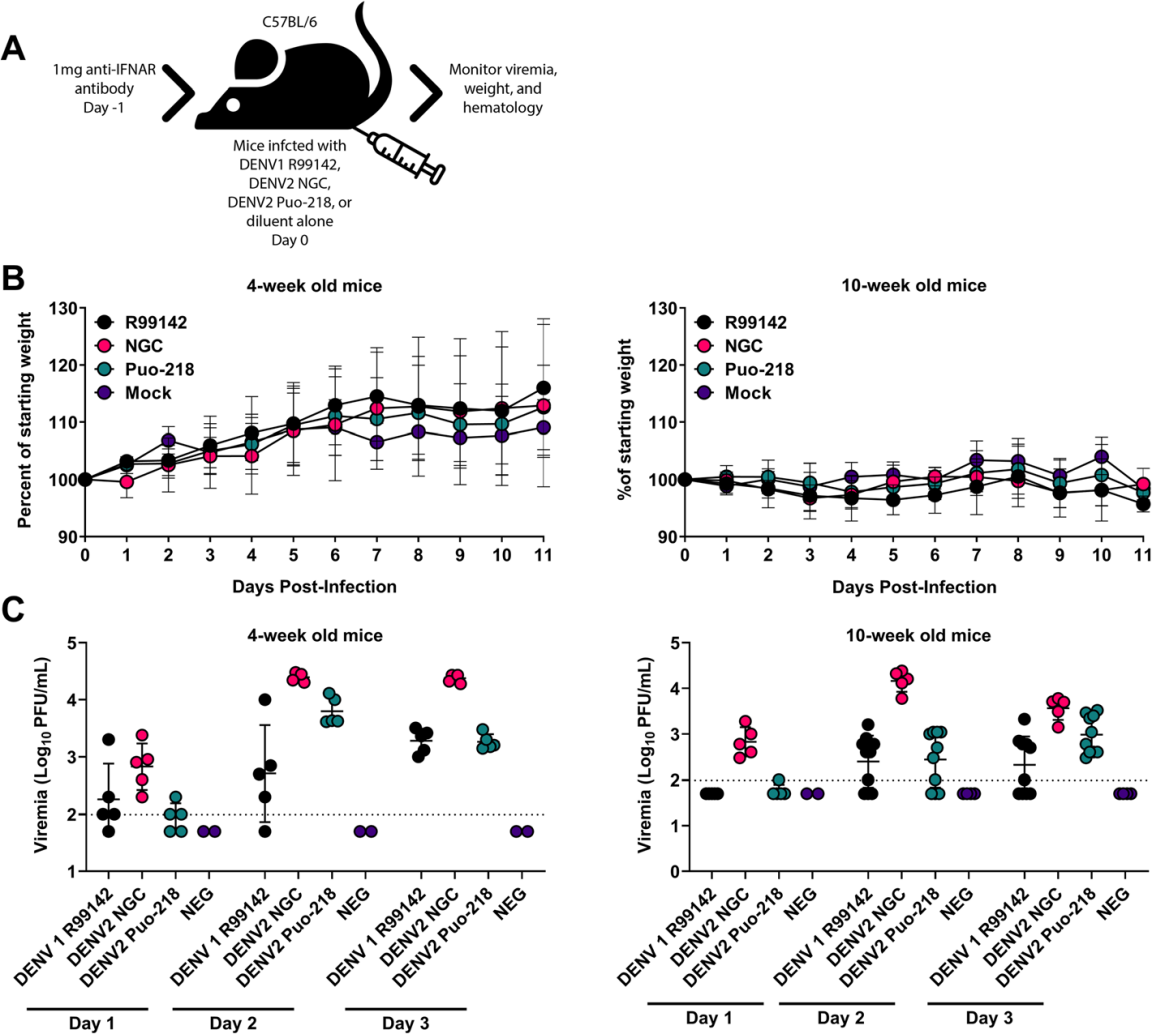
Dengue virus (DENV) replication in wild-type immunocompetent mice treated with interferon receptor blocking antibody. **a** Four-week- or 10-week-old female C57BL/6 J mice were treated with 1 mg of antibody to block interferon receptor signaling (IFNAR blocking antibody) and then infected with either DENV1 R99142, DENV2 NGC, or DENV2 Puo-218. **b** Percent weight loss throughout the study following infection. **c** Viremia following infection as determined by plaque assay in Vero cells. Values are means ± SD from groups of 5–10 animals, except for the mock, which had 2 animals. The studies in 4-week-old mice were repeated only once. The studies in 10-week-old mice were repeated twice, except for the DENV2 NGC groups, which was performed once. Data from the two biological replicates were combined for the 10-week-old mice. No statistical comparisons were made. The dotted line represents the limit of detection (LOD); all negative samples were given a value of 0.5 × LOD for statistical purposes

The goal of these studies was to identify low-passage DENV1 and DENV2 strains for use in studies assessing the influence of nutritional status on DENV infection. We selected one strain of DENV1 and two strains of DENV2 based on the time in which plaques formed on Vero cells and their passage history. The day before infection, we treated mice with 1 mg of IFNAR blocking antibody to increase susceptibility, as was previously done for West Nile virus and Zika virus [15, 16]. We infected 4-week- and 10-week-old mice with three DENV strains: DENV1 R99142, DENV2 NGC, and DENV2 Puo-218. DENV1 R99142 and DENV2 Puo-218 are relatively low-passage isolates, having undergone 3 and 5 passages, respectively. DENV2 NGC was passaged at least 19 times before use. Following infection, we measured viremia daily for 3 days and weights for 11 days (Fig. 1a). Neither 4-week- or 10-week-old mice experienced significant weight loss following infection with any DENV strain (Fig. 1b). All mice, regardless of age or virus, developed viremia after infection (Fig. 1c). Mice infected with DENV2 NGC had higher levels of viremia in both 4-week- and 10-week-old mice (*p* < 0.05 for all comparisons to both DENV1 R99142 and DENV2 Puo-218 on day 2 post-infection). Since DENV1 R99142 and DENV2 Puo-218 were low-passage isolates that produced viremia, we chose to use these viruses for all future infection studies and use the more virulent DENV2 NGC as a challenge virus in future studies.

**Fig. 2:**
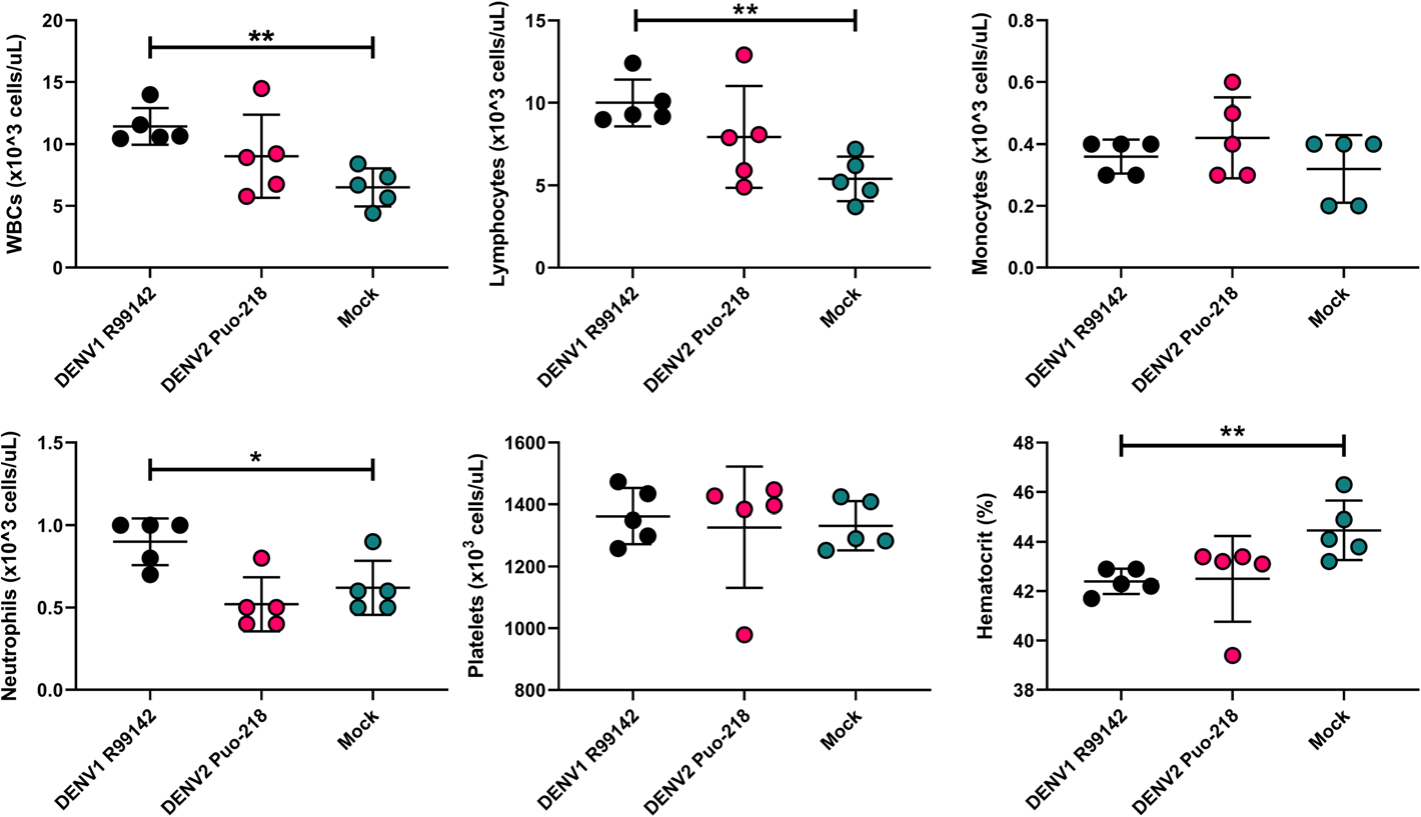
Hematological changes following dengue virus infection. Ten-week-old female C57BL/6 J mice were treated with 1 mg of antibody to block interferon receptor signaling (IFNAR blocking antibody) and then infected with DENV1 R99142 or DENV2 Puo-218. Hematological analysis was performed 7 days post-infection. Values are means ± SD from groups of 5 animals. Statistical comparisons were made to the mock group using one-way ANOVA with Dunnett’s comparison. *p < 0.05 and **p < 0.01. Studies were performed in one biological replicate

DENV infection in humans is associated with hematological changes, notably a reduction in platelets associated with the hemorrhagic disease. We, therefore, sought to assess the hematological outcomes in IFNAR blocking antibody-treated mice infected with DENV. Seven days post-infection (7 dpi), we collected blood samples from mice and submitted them for hematological assessment. No differences were observed between DENV2 Puo-218-infected mice and the mock-infected controls (Fig. 2). Compared to mock-infected controls, DENV1 R99142-infected mice displayed higher levels of white blood cells (WBCs, *p* < 0.0099), lymphocytes (*p* < 0.0089), neutrophils (*p* < 0.0283), and lower hematocrit (*p* < 0.0052). Platelet and monocyte levels were similar between all groups. Various other hematological parameters were unchanged following infection at 7 dpi, but some minor differences were observed at 13 dpi (Fig. S1 and S2).

### Previous DENV1 or DENV2 infection protects against challenge with a more virulent DENV2 strain

**Fig. 3:**
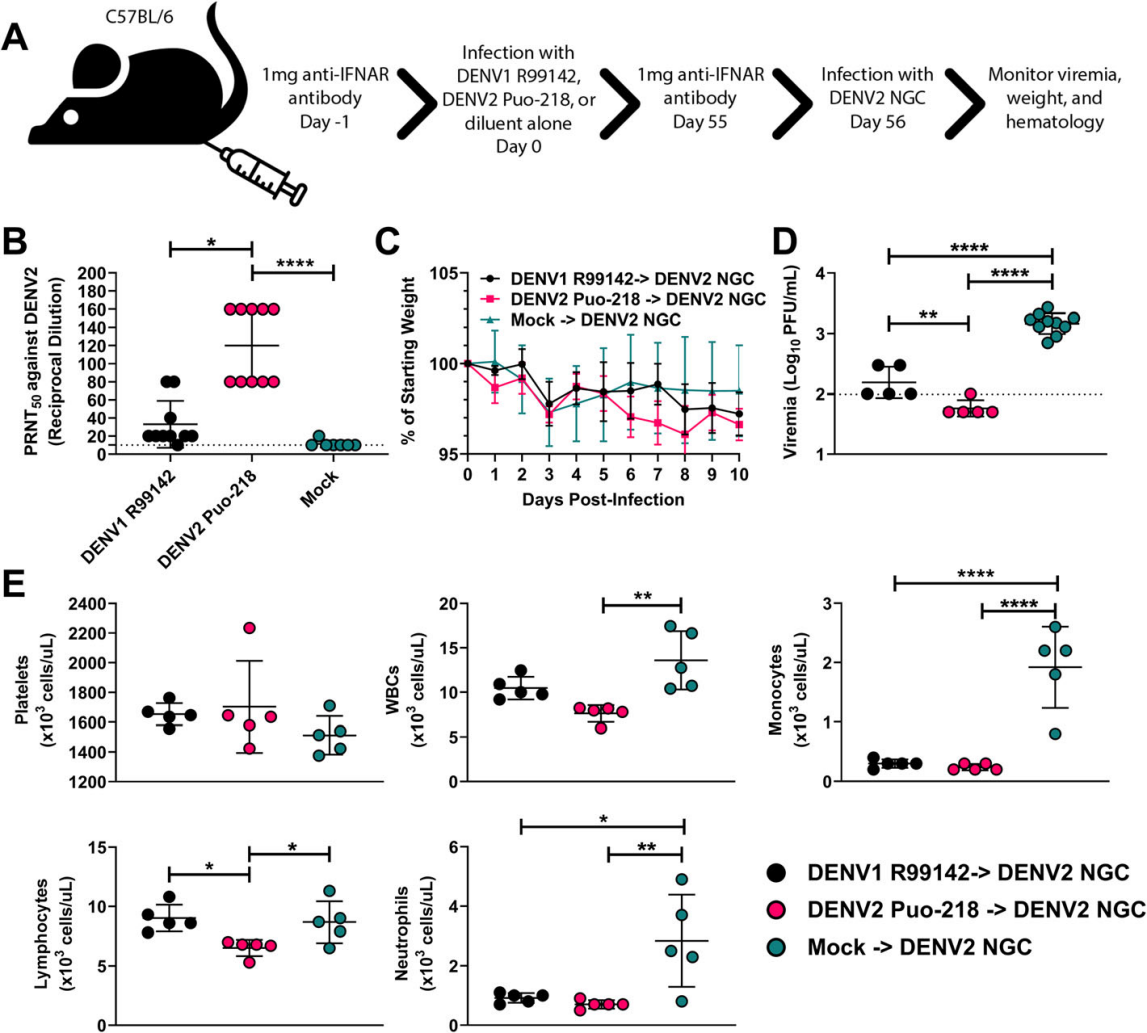
Protection mediated by a prior dengue virus infection on a heterologous or homologous challenge. **a** Six-week-old female C57BL/6 J mice were treated with 1 mg of antibody to block interferon receptor signaling (IFNAR blocking antibody) and then infected with DENV1 R99142 or DENV2 Puo-218. **b** Neutralizing antibodies measured by 50% plaque reduction neutralization test (PRNT_50_) against DENV2 Puo-218. Values are means ± SD from groups of 10 animals. **c**–**e** Fifty-five days later, mice were again treated with 1 mg of IFNAR blocking antibody and then infected with DENV2 NGC. **c** Percent weight loss throughout the study following infection. **d** Viremia 2 days following infection as determined by plaque assay in Vero cells. **e** Hematological analysis was performed 7 days post-infection. Values are means ± SD from groups of 5–10 animals. Statistical comparisons were made using a one-way ANOVA with Dunnett’s comparison for all panels except **c**, which was made to the mock group using a repeated measures mixed-effects model analysis with Dunnett’s correction. *p < 0.05, **p < 0.01, ****p < 0.0001. Studies were performed in one biological replicate, except for **b**, which was performed in two independent biological replicates. The dotted line represents the limit of detection (LOD); all negative samples were given a value of 0.5 × LOD for statistical purposes

In humans, previous DENV infection is associated with more severe disease following re-infection with a new serotype [17–19]. In contrast, infection with one serotype is thought to provide life-long protection against re-infection with the same serotype [20]. However, this dogma has been challenged recently in the field [21] and in vaccination studies [22, 23], where re-infections occurred despite the presence of neutralizing antibodies. To determine if a previous DENV1 or DENV2 infection provides sterilizing immunity to challenge with a virulent DENV2 challenge in the presence of neutralizing antibodies in our mouse model, we infected mice previously exposed to DENV1 R99142 or DENV2 Puo-218 with DENV2 NGC (Fig. 3a). Before challenge, mice previously infected with DENV1 R99142 had moderate levels of neutralizing antibodies against DENV2 Puo-218 (Fig. 3b, geometric mean titer (GMT) PRNT_50_ of 26.39) and 9/10 mice had detectable neutralizing titers. All of the mice previously infected with DENV2 Puo-218 seroconverted (GMT PRNT_50_ of 113.1), which was significantly higher than the mock-infected controls (GMT PRNT_50_ of 11.04, *p* < 0.0001 between the mock- and DENV2-infected groups). Neutralizing antibody titers against DENV2 were higher for DENV2-infected mice compared to DENV1-infected mice (*p* = 0.015).

Following challenge, no significant differences in weight loss were observed (Fig. 3c). Viremia at 2 dpi was significantly reduced in groups previously infected with DENV1 and DENV2 compared to mock-infected controls (Fig. 3d, *p* < 0.0001 for both comparisons). Viremia in mice previously exposed to DENV1 was higher than mice with a prior DENV2 infection (*p* = 0.0058). While 5/5 mice previously infected with DENV1 had detectable viremia at 2 dpi, only 1/5 mice with prior DENV2 exposure had viremia detectable by plaque assay. Levels of platelets were unaffected in any group following challenge (Fig. 3e). Mice with prior DENV2 exposure had reduced WBC (*p* = 0.0020), monocyte (*p* < 0.0001), lymphocyte (*p* = 0.048), and neutrophil counts (*p* = 0.0071) at 7 dpi compared to mock-infected controls. Mice previously infected with DENV1 had lower monocyte (*p* < 0.0001) and neutrophil counts (*p* = 0.014) compared to mock-infected controls. Lymphocyte counts were lower in mice previously infected with DENV2 compared to DENV1-infected mice (*p* = 0.023).

### Nutritional status alters disease severity in DENV2-infected mice

**Fig. 4:**
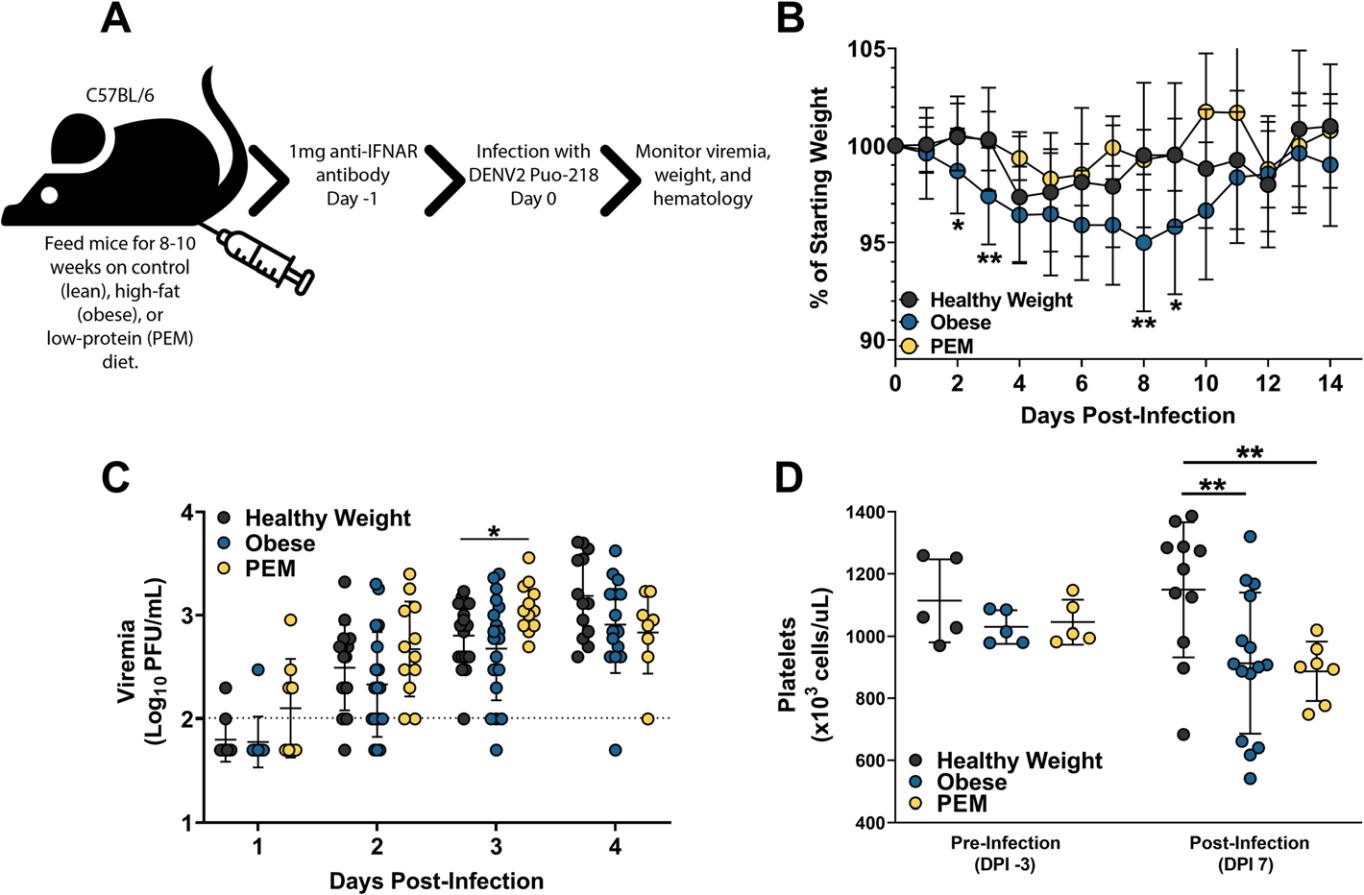
Nutritional status alters dengue virus (DENV) infection. **a** Six-week-old female C57BL/6 J mice were fed for 8–10 weeks on a control (healthy weight), high-fat (obese), or low-protein (protein-energy malnutrition, PEM) diet and then treated with 1 mg of antibody to block interferon receptor signaling (IFNAR blocking antibody). The next day, mice were infected with DENV2 Puo-218. **b** Percent weight loss throughout the study following infection. **c** Viremia following infection as determined by plaque assay in Vero cells. The dotted line represents the limit of detection (2.1 Log_10_ PFU/mL); all points below this line represent samples with no plaques, which we gave an arbitrary value of half of the limit of detection (1.8 Log_10_ PFU/mL). **d** Levels of platelets 7 days post-infection. Values are means ± SD from groups of 7–19 animals combined from 2 to 3 independent biological replicates, except for the pre-infection platelet levels, which were measured only once. Statistical comparisons were made to the healthy weight group using a repeated measures mixed-effects model analysis with Dunnett’s correction. *p < 0.05, **p < 0.01

Epidemiological studies in humans have identified an association between nutritional status and disease outcome [6–8]. To test this, we fed groups of mice either a control (herein referred to as healthy weight), high-fat (herein referred to as obese), or low-protein (herein referred to as PEM) diet for 8–10 weeks. Following the feeding period, mice were treated with 1 mg of IFNAR blocking antibody and then infected with DENV2 Puo-218 (Fig. 4a). Obese mice weighed significantly more than healthy weight controls at the time of infection, while no difference was observed between healthy weight and PEM mice (Fig. S3). Following infection, obese mice experienced significantly higher weight loss compared to healthy weight controls (Fig. 4b, *p* < 0.05 at 2 dpi and 9 dpi and *p* < 0.01 at 3 dpi and 8 dpi). Replication kinetics were similar between all groups, and the only significant difference observed was at 3 dpi between healthy weight and PEM mice (Fig. 4c, *p* = 0.03). We also tested organs from infected mice at 3 dpi to assess viral dissemination; while 4/4 mice in both the obese and PEM groups contained infectious virus in the footpad, only 2/4 were virus-positive for the healthy weight group (Fig. S4). Only the PEM group had infectious virus in the spleen at 3 dpi (*p* = 0.0015 compared to healthy weight controls), while no infectious virus was detected in the liver for any group at this time point. Thrombocytopenia is a crucial indicator of dengue disease severity [24]. Accordingly, we measured the level of platelets and other hematological markers in infected mice at 7 dpi. Both obese and PEM mice had significantly lower platelets compared to healthy weight controls (Fig. 4d, *p* = 0.0125 and 0.0235 for obese and PEM, respectively). No significant differences were observed for any other hematological parameters tested (Fig. S5).

Severe DENV disease is associated with high levels of pro-inflammatory cytokines. To determine if altered cytokine expression could explain the increased disease severity observed in obese mice, we assessed the serum levels of 48 cytokines simultaneously using a multiplex Luminex cytokine panel. We tested only healthy weight and obese samples in these studies since disease severity in terms of weight loss and thrombocytopenia was the highest in obese mice (Fig. 4). We observed no differences between pre- and post-infection levels for any cytokines tested for healthy weight mice (Table 1). However, following infection, obese mice had increased circulating levels of B cell activating factor (BAFF, *p* = 0.0070), CCL5 (*p* = 0.0118), CCL17 (*p* = 0.0118), Chitinase-3-like 1 (*p* = 0.0118), CXCL5 (*p* = 0.0118), and IFN-alpha (*p* = 0.0118). No differences in any cytokine were observed when healthy weight and obese mice were compared directly (Supplemental Table 2). These data indicate that with our mouse model, cytokine expression is altered following DENV infection in obese, but not healthy weight mice.

**Table 1:**
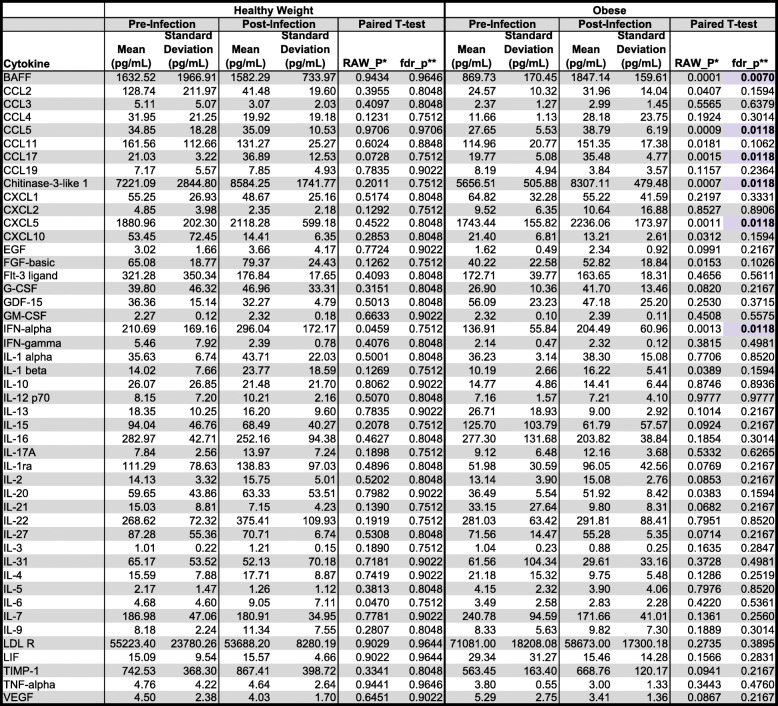
Comparison of cytokine levels pre- and post-dengue virus infection in healthy weight and obese mice

*RAW_P indicates the *p* value from a paired *t* test comparing pre-infection and post-infection values

**fdr_p indicates *p* values adjusted for multiple testing using the Benjamini-Hochberg false discovery rate method. Significant values are presented as bolded values highlighted in purple. Values presented are from five mice from two independent replicates

### Nutritional status impacts DENV tissue pathology

**Fig. 5:**
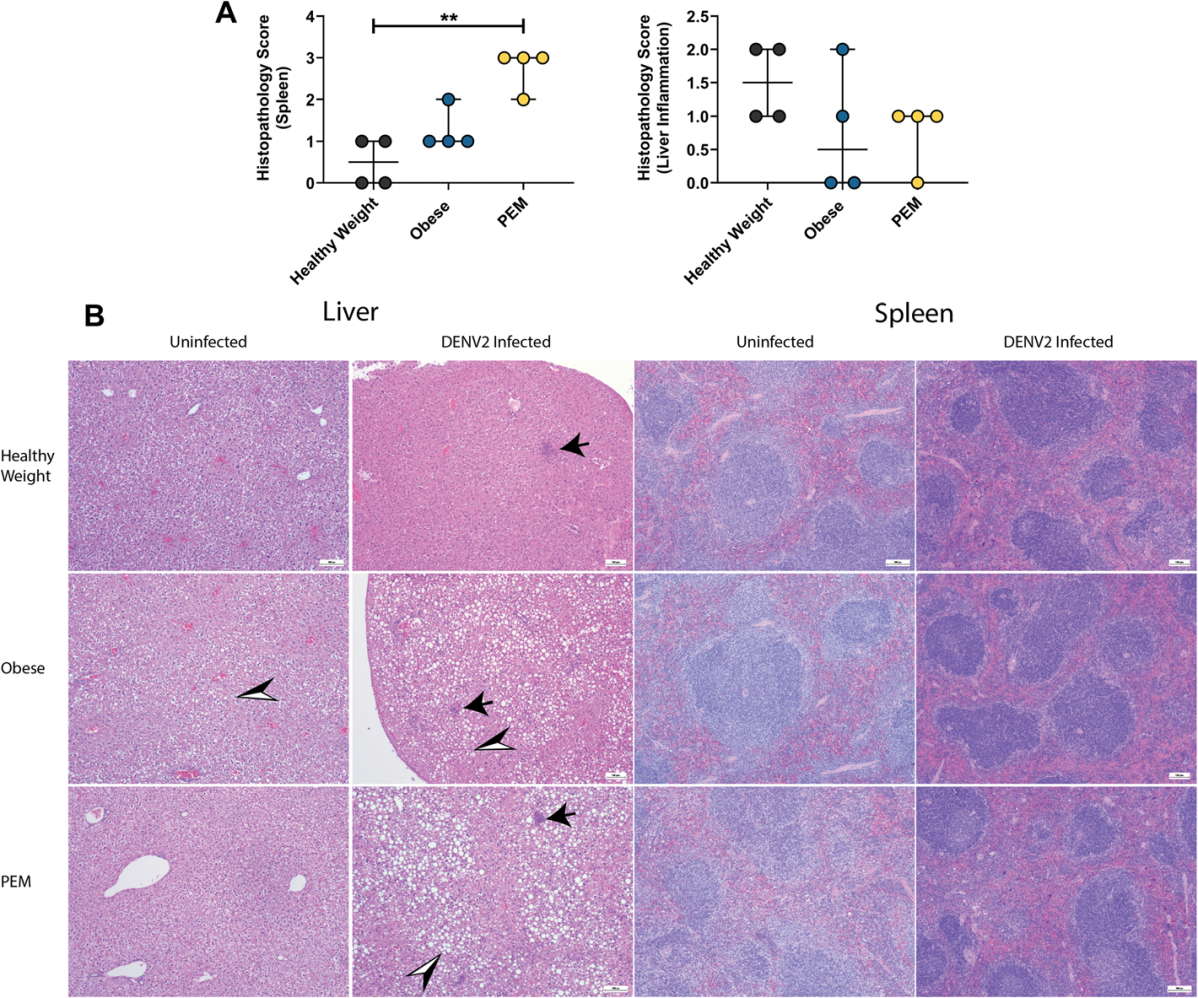
Histopathology following infection with dengue virus (DENV) in mice with varying nutritional status. Six-week-old female C57BL/6 J mice were fed for 8–10 weeks on a control (healthy weight), high-fat (obese), or low-protein (protein-energy malnutrition, PEM) diet and then treated with 1 mg of antibody to block interferon receptor signaling (IFNAR blocking antibody). The next day, mice were infected with DENV2 Puo-218. **a** Histology scores from the spleen and liver collected 2 days post-infection. Values are presented as medians with the minimum and maximum of the range from groups of four animals. Statistical comparisons were made using the Kruskal-Wallis test with Dunn’s multiple comparisons test. *p < 0.05 and **p < 0.01. **b** Representative images of the liver and spleen collected before and after infection with DENV2. Black-filled arrows represent areas of inflammation. Black and white arrows represent areas of lipidosis. Scale bar, 100 μM. Data presented are from one biological replicate

Dengue disease can result in splenic rupture [25–27] or severe liver inflammation [25]. To determine the role of nutritional status on DENV pathology, we euthanized mice at 2 dpi, and a board-certified anatomic pathologist scored the slides in a blinded manner. PEM resulted in more significant spleen inflammation and lymphoid follicular hyperplasia following DENV infection (Fig. 5a). We observed no differences in liver inflammation at this time point for any group. Representative images of the spleen and liver are presented in Fig. 5b.

### Malnourishment reduces heterologous protection despite similar neutralizing antibody titers

**Fig. 6:**
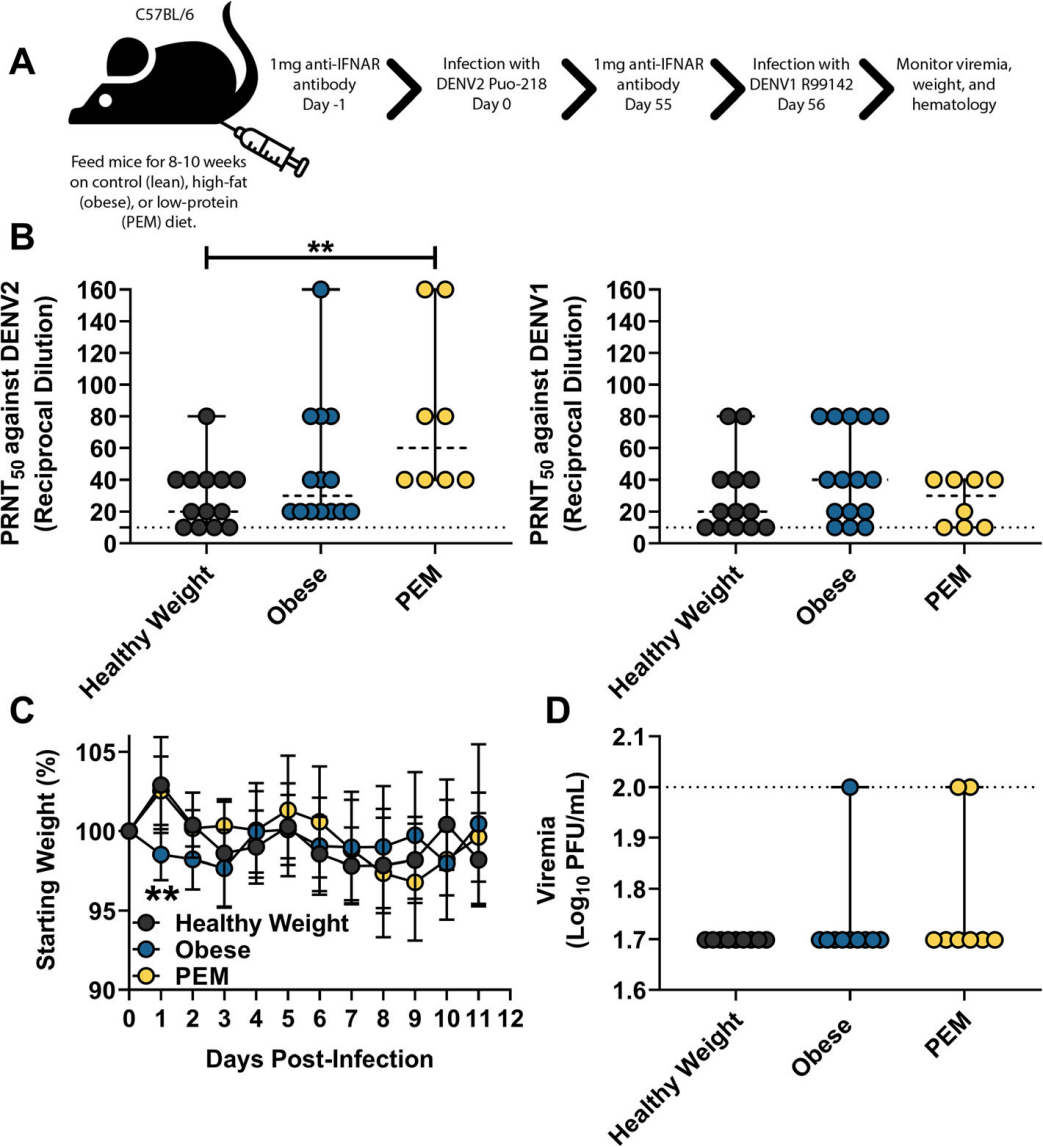
The influence of nutritional status on a secondary dengue virus (DENV) infection with another serotype. **a** Six-week-old female C57BL/6 J mice were fed for 8–10 weeks on a control (healthy weight), high-fat (obese), or low-protein (protein-energy malnutrition, PEM) diet and then treated with 1 mg of antibody to block interferon receptor signaling (IFNAR blocking antibody). The next day, mice were infected with DENV2 Puo-218. Values are means ± SD from groups of 8–10 animals from two biological replicates. **b** Neutralizing antibodies measured by 50% plaque reduction neutralization test (PRNT_50_) against DENV2 Puo-218 and DENV1 R99142. Values are means ± SD from groups of 8–14 animals. **c**, **d** Fifty-five days later, mice were again treated with 1 mg of IFNAR blocking antibody and then infected with DENV1 R99142. **c** Percent weight loss throughout the study following infection. Statistical comparisons were made to the healthy weight group using a repeated measures mixed-effects model analysis with Dunnett’s correction**.** Values are means ± SD. **d** Viremia following infection as determined by plaque assay in Vero cells. For **b** and **d**, values are presented as medians with the minimum and maximum of the range and statistical comparisons were made against the healthy weight group using the Kruskal-Wallis test with Dunn’s multiple comparisons test. **p < 0.01. The dotted line represents the limit of detection (LOD); all negative samples were given a value of 0.5 × LOD for statistical purposes

In our previous studies, we showed that mice previously infected with one DENV serotype were afforded partial protection with another serotype (Fig. 3). To evaluate the role of nutritional status on protection against challenge with a heterologous serotype, we challenged mice previously infected with DENV2 Puo-218 with DENV1 R99142 (Fig. 6a). We chose DENV2 for the initial infection since it is associated with highest disease severity in humans [28]. Before challenge, the levels of neutralizing antibodies were highest against DENV2 for the PEM group (Fig. 6b, *p* = 0.0068 compared to healthy weight mice). However, no differences were observed for neutralization against DENV1 for any group. Following challenge, obese mice lost significantly more weight at 1 dpi (Fig. 6c, *p* = 0.0042) compared to healthy weight controls, but they recovered quickly, and no further differences were observed. We found that 1/10 obese and 2/8 PEM mice had detectable viremia at 2 dpi, while no infectious virus was observed in healthy weight mice (Fig. 6d). Few hematologic differences were observed following challenge except for red blood cell distribution width or RDW-CV, which was reduced in obese mice (*p* = 0.0274) and increased in PEM mice (*p* = 0.0026) compared to healthy weight controls (Fig. S6).

## Discussion

Worldwide, it is estimated that 390 million people are infected annually with DENV, which can produce severe, sometimes deadly disease [1]. Given the prevalence of DENV, identifying risk factors for severe disease is critical. Nutritional status, both obesity and undernutrition, has been associated with altered disease severity following DENV infection [29]. However, no laboratory studies have directly shown that nutritional status alters DENV disease severity. Given the prevalence of both DENV infection and undernutrition/obesity, a small animal model to study the role of nutritional status on DENV and other arbovirus infections is necessary to provide insight on disease severity, transmission, vaccine efficacy, and pathogenesis.

We first sought to develop a mouse model of mild DENV infection with reliable viremia for use in studies to assess the role of nutritional status on viral replication, immune protection, transmission, and disease severity. We used low-passage isolates of DENV1 and DENV2 to more closely simulate the strains circulating in humans. Other groups have developed mouse-adapted strains [30, 31], which are useful for studying some aspects of severe DENV disease but may not reflect the natural pathogenesis of the parental virus. While the use of fully immunocompetent mice is optimal, this possibility is limited by their resistance to DENV replication. Several groups have used immunocompetent C57BL/6 [32], A/J [33, 34], or BALB/c [35] to produce various disease manifestations, but none result in detectable infectious virus, making it impossible to study transmission or viral evolution. In these studies, we used C57BL/6 mice transiently rendered susceptible to infection by treatment with IFNAR blocking antibody as was previously described for WNV [15] and ZIKV [16]. Following infection with low-passage DENV1 or DENV2, mice developed viremia that was detectable by plaque assay for several days but caused no overt disease, consistent with previous experiments in IFNAR blocking antibody-treated mice infected with ZIKV [16]. Many previously described DENV mouse models are highly immunocompromised, lacking type-1 IFN receptors [31, 36] or both type-1 and type-2 IFN receptors [30, 37]. While these models are useful for studying severe DENV disease, they result in high levels of mortality, which is a relatively rare manifestation in humans. Furthermore, for vaccination studies, the use of a transiently immunocompromised mouse model allows for a natural immune response upon vaccination, which might be more clinically relevant in comparison to mice with genetic deletions in the interferon system.

While most DENV mouse models mimic severe disease, our model more closely represents an inapparent DENV infection, which is meaningful since the vast majority of DENV infections produce no apparent disease. Rates of inapparent DENV infection vary between outbreaks. Ranges from 11 to 88% have been observed [38], and some believe that this is an underappreciated concern [39, 40]. Developing a model for inapparent DENV is significant since the contribution to transmission is likely high, and the probability of severe disease increases upon infection with a second serotype. In these studies, mice infected with DENV1 R99142 or DENV2 NGC, and to a lesser extent DENV2 Puo-218, developed leukocytosis, lymphocytosis, neutrophilia, and decreased hematocrit. Lymphocytosis is characteristic of acute symptomatic DENV infection; however, typically, leukopenia, neutropenia, and increased hematocrit are observed [41]. Little is known about hematological parameters following inapparent DENV infection, although leukocytosis with neutrophilia has been seen early in DENV infection [42]. In 1952, Sabin described a mild type of DENV with fever lasting from 24 to 36 h with no rash or leukocytosis [43]. While the relevance of the hematological changes we observed is unclear, it highlights the need for studies on inapparent DENV infections, possibly in human volunteers [44].

To determine if our model could be used for protection studies, we challenged mice previously infected with DENV1 R99142 or DENV2 Puo-218 with DENV2 NGC, a more virulent strain in these mice. After only 56 days, 100% of the mice previously infected with DENV1 were susceptible to DENV2 infection, as judged by detectable viremia, albeit to lower levels than the control group, as has previously been observed [45]. The hematological changes induced by DENV2 NGC (Fig. 3e) were similar to those observed following DENV1 R99142 infection (Fig. 2); this may indicate that these viruses are more virulent than DENV2 Puo-218. Mice previously infected with DENV1 R99142 or DENV2 Puo-218 were protected from leukocytosis, monocytosis, and neutrophilia following DENV2 NGC challenge. Only DENV2 Puo-218 immune mice, however, were protected from lymphocytosis, possibly indicating a key role for lymphocytes in cross-reactive DENV immunity in this model. These data suggest that this mouse model could be useful for studying heterologous immunity, particularly if the mice are challenged after a longer duration from the primary infection. This model could be useful for vaccination studies since the challenge can be performed with 2 DENV serotypes, and viremia would be an indicator of sterilizing immunity. Since mice survive DENV infection, our model can be used to assess vaccine efficacy in both DENV-naive and DENV-immune individuals.

We then applied this model to study the effect of nutritional status on DENV infection and found that obese mice experience significantly higher morbidity than healthy weight controls in terms of weight loss and thrombocytopenia, a key marker of severe DENV disease [46]. These results are consistent with several epidemiological studies, which suggested that obese individuals have an increased risk for severe disease [10, 11, 47]*.* We also observed that obesity increased serum levels of several cytokines following DENV2 infection. In contrast, no changes for any cytokine were observed in healthy weight mice following DENV infection. Obesity substantially increased serum BAFF, which facilitates B cell survival and differentiation [48, 49]. BAFF levels positively correlate with DENV viremia in humans [46] and are significantly increased following DENV infection of endothelial cells [50]. Kwissa et al. found that CD14+CD16+ monocytes secrete BAFF following DENV infection, resulting in the differentiation of resting B cells to plasmablasts [51]. Since obesity alters the types of immune cells circulating [52], along with their activation status [53, 54], more CD14+CD16+ monocytes may be present after infection of an obese host as compared to a healthy weight host. We observed similar neutralizing antibody response between obese and healthy weight mice; accordingly, the relevance for the increase in BAFF is unclear. BAFF is secreted by adipocytes [55] and regulates systemic inflammation in obese mice [56]. The data presented here—along with the existing literature—indicate that BAFF may be a driver of dengue disease severity.

Obese mice also had increased serum levels of CCL5 (RANTES), CCL17, CXCL5 (LIX), chitinase-3-like 1, and IFN-alpha in our mouse model following DENV infection as compared to before (Table 1). Guabiraba et al. observed increased CCL5 and CCL17 expression in mice [57], and the same group found that mice lacking CCR5—the receptor for CCL5, CCL3, and CCL4—were protected from DENV-induced mortality [58]. DENV-infected people had high levels of CCL5 and chitinase-3-like 1 during acute infection [59, 60], and livers from fatal dengue patients had increased CCL5 compared to non-dengue control [61]. Additionally, human microvascular endothelial cells produce CCL5 and CXCL5 following DENV infection, which could contribute to vascular leakage and severe disease [62]. Finally, the increase in IFN-alpha could explain the weight loss observed in obese mice following infection, since it is known to have an anorexic effect [63]. The increase in these cytokine levels following DENV infection likely contributed to the weight loss and reduced platelet levels we observed in obese mice.

In these studies, we used only female mice and an obesogenic diet containing 45% (saturated) fat. Other groups have used male mice with a 60% fat diet to generate more obese mice. Accordingly, the differences in weight between the obese and healthy weight groups were not as dramatic as has been previously observed [64, 65]. The use of a more obesogenic diet for a more extended period might result in further increases in disease severity and will be implemented in future studies. However, our results suggest that even mild obesity can result in increased disease severity and, as such, should be considered a risk factor for the development of severe DENV disease.

PEM mice did not lose weight following infection but did have reduced platelet levels compared to controls. Furthermore, PEM mice had increased spleen pathology and higher viral titers in the spleen following infection. These results were surprising since undernourishment has been associated with protection from severe DENV disease by several groups [6–8, 11]. However, one group did find that undernourished patients had a higher risk of developing dengue shock syndrome (DSS) [11]. Spleen pathology has been reported following DENV infection and is mostly associated with severe DENV disease resulting in splenic rupture [25–27]. The relevance of the increased spleen pathology observed here is unclear since mice produced no overt symptoms following infection. We previously found increased disease severity in PEM mice infected with chikungunya virus or Mayaro virus [66], suggesting that this observation may be true for many viral infections. The results of our studies are tempered by the existence of many forms of undernutrition, including PEM, but also micronutrient deficiencies, underweight, wasting, and stunting [67]. Accordingly, our conclusions are limited to PEM. In these studies, we used a PEM diet containing 5% protein, while other groups have used diets containing as little as 2% protein [14, 68]. Since estimates suggest that protein consumption in forty sub-Saharan African countries ranged from 6.1 to 13.5% [69], our use of 5% appears to reflect what is observed naturally.

Obese humans or mice infected with or vaccinated against influenza have reduced vaccine efficacy and lower adaptive immune responses [12, 13, 70–73]. Similarly, PEM reduces adaptive immune response in mice [14, 74] but appears to have a minor effect in humans [75]. Given the importance of pre-existing immunity upon secondary infections in DENV disease, we sought to assess the influence of nutritional status on protection against a challenge with a second serotype. While we observed high levels of protection in all groups, obese mice lost weight early post-challenge, and the only mice to develop viremia were in the obese and PEM groups. In these studies, we challenged the mice 56 days following the primary infection, which may have been insufficient time for cross-protective immunity to wane. Conclusions based on these data must be tempered by the small differences observed between the groups; future studies should use a more robust challenge and a more prolonged time post-primary infection to assess differences in protection following secondary infection.

The mechanisms underlying the differences observed in pathology between obese/PEM and healthy weight mice are unknown. Diet significantly impacts immunity, metabolic status, and the microbiome, among other things. We found some evidence that obesity alters the immune response to DENV infection, which likely contributes to the differences in morbidity observed. Both obesity and PEM dramatically shift the proportion of immune cells present and their functional status [75, 76]. For example, macrophages comprise roughly 40% of all cells in adipose tissue, while PEM lowers total levels of lymphocytes [76, 77]. Obesity and PEM also alter metabolic parameters that can influence DENV infection: for example, glucose, insulin, lipids, micronutrients, and many more factors [78–87]. Finally, the microbiome plays a critical role in responding to pathogens. A recent report showed that a high-fiber diet increased levels of microbiome-induced short-chain fatty acid (SCFAs), resulting in more severe CHIKV disease [88]. Currently, there is a lack of studies identifying the influence of the microbiome on DENV infection, which is a critical gap in the literature. Taken together, the complexity of nutritional status leaves many open questions relating to how disease severity can be altered following infection.

## Conclusions

In summary, we present empirical evidence that nutritional status alters DENV disease severity, consistent with previously reported epidemiological evidence. We describe a mouse model for inapparent DENV infection with consistent viremia levels that is useful for nutritional studies and vaccination experiments. Our results demonstrate that obesity and, to a lesser extent, PEM result in more severe disease following DENV infection. This work, along with others, suggests that nutritional status should be considered in public health strategies aimed at controlling severe viral disease.

## Methods

### Viruses and cells

All DENV strains used in these studies were obtained from the Centers for Disease Control and Prevention (CDC). DENV1 strain R99142 was isolated in 2013 from a traveler to Guatemala and was passaged once in Vero cells and twice in C6/36 cells (once at the CDC and once in our lab). DENV2 strain Puo-218 was isolated in 1980 from a human in Thailand and was passaged once in Vero cells and four times (three times at the CDC and once in our lab) in C6/36 cells. DENV2 strain New Guinea C (DENV2 NGC) was isolated in 1944 from a human in New Guinea and underwent 17 passages in an unknown host, followed by 2 passages in C6/36 cells (once at the CDC and once in our lab). The passaging history for all viruses tested was provided by the CDC. C6/36 and Vero cells were maintained in Dulbecco’s modified Eagle’s medium (DMEM) with 5% fetal bovine serum (FBS), non-essential amino acids, and gentamicin.

### Mouse experiments

Female C57BL/6 J mice were obtained from The Jackson Laboratory. All studies were performed in an approved animal biosafety level 2 (ABSL2) facility. Twenty-four hours before infection, mice were given 1 mg of MAR1-5A3, an anti-interferon-α/β receptor (IFNAR) antibody (Leinco Technologies, herein referred to as IFNAR blocking antibody) intraperitoneally (i.p.). Mice were infected via a combined intradermal (i.d.)/subcutaneous (s.c.) route by injecting 50 μL into each footpad and 100 μL under the skin of the back. We used this method of injection to maximize the viral dose that could be given to the mice. The virus was diluted in Roswell Park Memorial Institute (RPMI) 1640 (RPMI-1640) containing 1% FBS (herein called viral diluent). Blood was collected into a serum separation tube for viremia measurement and frozen before testing by plaque assay. Tissues for viral titration were homogenized in viral diluent using a TissueLyser II (QIAGEN) for 2 min at 30 cycles per second. Plaque assays were then performed on the cleared homogenate. Following infection, weights were measured daily for each mouse.

### Cytokine measurements

Serum cytokines were measured using the Luminex XL Cytokine Mouse Kit (Bio-Techne). Serum samples were diluted 1:1 according to the manufacturer’s protocol, and the levels of cytokine were fit to a seven-point standard curve. Data was collected using the Luminex FLEXMAP 3D system.

### Hematology and histopathology

Hematological parameters were assessed by Virginia Tech Animal Laboratory Services (ViTALS), an American Association of Veterinary Laboratory Diagnosticians (AAVLD)-accredited diagnostic laboratory. For histopathology, formalin-fixed tissues were prepared by ViTALS, and the slides were read by a board-certified anatomic pathologist (TL). The scores refer to the extent of lymphoid follicular hyperplasia in the spleen and inflammation in the liver.

### Diets and feeding experiments

All diets used in these studies were obtained from Envigo. The composition of each diet is presented in Supplemental Table 1. For nutritional studies, mice were obtained at 4–6 weeks of age and allowed to feed for 8–10 weeks. The diet was maintained for the entire duration of the studies, including after infection. Throughout the manuscript, we will refer to the groups as follows: control diet (healthy weight), high-fat diet (obese), and 5% protein diet (protein-energy malnutrition or PEM). Mice were weighed weekly before infection.

### Plaque assays and plaque reduction neutralization tests (PRNTs)

Plaque assays and PRNTs were performed in Vero cells. For plaque assays, samples were serially diluted tenfold in viral diluent. For PRNTs, twofold dilutions of serum were performed in viral diluent that was then mixed with virus at 800 PFU/mL, which was then incubated at 37 °C for 1 h to allow neutralization to occur. For both assays, 50 μL was then added to a well of a 24-well plate and allowed to incubate at 37 °C for 1 h. Overlay media containing 0.6% tragacanth gum, 1× MEM, 20 mM HEPES, and 4% FBS were then added, and the plates were allowed to incubate at 37 °C to allow plaques to form.

### Statistical analysis

All statistical analyses were performed in GraphPad version 8. One- and two-way ANOVAs with Dunnett’s multiple comparisons test were used for most tests. A *p* value of less than 0.05 was considered significant. For viremia graphs, the dotted line represents the limit of detection (2.1 Log_10_ PFU/mL); all points below this are samples with no plaques observed, which we gave an arbitrary value of half of the limit of detection (1.8 Log_10_ PFU/mL). Pre- and post-infection cytokine concentrations were compared using a paired *t* test. Comparisons between the healthy weight and obese groups were made using an unpaired *t* test. *p* values were adjusted for multiple testing using the Benjamini-Hochberg false discovery rate method.

## Supplementary information

**Additional file 1: Figure S1.** Hematological changes following dengue virus infection. 10-week old female C57BL/6J mice were treated with 1 mg of antibody to block interferon receptor signaling (IFNAR blocking antibody) and then infected with DENV1 R99142 or DENV2 Puo-218. Hematological analysis was performed 7 days post-infection. Values are means ± SD from groups of 5 animals. Statistical comparisons were made to the mock group using one-way ANOVA with Dunnett’s comparison. Studies were performed in one biological replicate.

**Additional file 2: Figure S2.** Hematological changes following dengue virus infection. 10-week old female C57BL/6J mice were treated with 1 mg of antibody to block interferon receptor signaling (IFNAR blocking antibody) and then infected with DENV1 R99142 or DENV2 Puo-218. Hematological analysis was performed 13 days post-infection. Values are means ± SD from groups of 5 animals. Statistical comparisons were made to the mock group using one way ANOVA with Dunnett’s comparison. * indicates p<0.05. Studies were performed in one biological replicate.

**Additional file 3: Figure S3.** Weight during feeding before infection. 6-week old female C57BL/6J mice were fed for 8-10 weeks on a control (healthy weight), high-fat (obese), or low-protein (protein-energy malnutrition, PEM) diet. The mice were weighed weekly during feeding. Values are means ± SD from groups of 12-19 animals combined from three independent studies. For studies with a time component, statistical comparisons were made with a two way ANOVA while AUC comparisons were made using a one-way ANOVA, both used Dunnett’s multiple comparisons test to the healthy weight group. * indicates p<0.05, ** indicates p<0.01.

**Additional file 4: Figure S4.** Organ titers in mice with different nutritional status following dengue virus (DENV) infection. 6-week old female C57BL/6J mice were fed for 8-10 weeks on a control (healthy weight), high-fat (obese), or low-protein (protein-energy malnutrition, PEM) diet and then treated with 1 mg of antibody to block interferon receptor signaling (IFNAR blocking antibody). The next day, mice were infected with DENV2 Puo-218, and three days later, the mice were euthanized, and tissues were collected. Tissue viral load was determined by plaque assay in Vero cells. Values are means ± SD from groups of 4 animals. Statistical comparisons were made to the mock group using one-way ANOVA with Dunnett’s comparison. Studies were performed in one biological replicate. ** indicates p<0.01. The dotted line represents the limit of detection (LOD); all negative samples were given a value of 0.5 x LOD for statistical purposes.

**Additional file 5: Figure S5.** Hematological changes following dengue virus infection in mice with different nutritional status. 6-week old female C57BL/6J mice were fed for 8-10 weeks on a control (healthy weight), high-fat (obese), or low-protein (protein-energy malnutrition, PEM) diet and then treated with 1 mg of antibody to block interferon receptor signaling (IFNAR blocking antibody). The next day, mice were infected with DENV2 Puo-218, and hematological analysis was performed 7 days later. Values are means ± SD from groups of 7-15 animals. Statistical comparisons were made to the mock group using one-way ANOVA with Dunnett’s comparison. Studies were performed in two biological replicates.

**Additional file 6: Table S2.** Comparison of cytokine levels between healthy weight and obese mice pre- and post-dengue virus infection.

**Additional file 7: Figure S6.** Hematological changes following secondary dengue virus infection in mice with different nutritional status. 6-week old female C57BL/6J mice were fed for 8-10 weeks on a control (healthy weight), high-fat (obese), or low-protein (protein-energy malnutrition, PEM) diet and then treated with 1 mg of antibody to block interferon receptor signaling (IFNAR blocking antibody). The next day, mice were infected with DENV2 Puo-218. Fiftyfive days later, mice were again treated with 1 mg of IFNAR blocking antibody and then infected with DENV1 R99142. Hematological analysis was performed 7 days post-infection. Values are means ± SD from groups of 4-5 animals. Statistical comparisons were made to the mock group using one-way ANOVA with Dunnett’s comparison. Studies were performed in one biological replicate. * indicates p<0.05. ** indicates p<0.01.

**Additional file 8: Supplementary Table 1.** Composition of diets used in feeding experiments.

## Data Availability

The datasets used and/or analyzed during the current study are enclosed in the manuscript or available from the corresponding author upon request.

## Abbreviations

DENV: Dengue virus
DENV1: Dengue virus serotype 1
DENV2: Dengue virus serotype 2
PEM: Protein-energy malnutrition
PRNT: Plaque reduction neutralization test
