# Supplementary figures and images for: Nutritional status impacts dengue virus infection in mice
Source: BMC Biol. 2020 Aug 27;18:106. doi: 10.1186/s12915-020-00828-x (PMC7453574; doi:10.1186/s12915-020-00828-x)

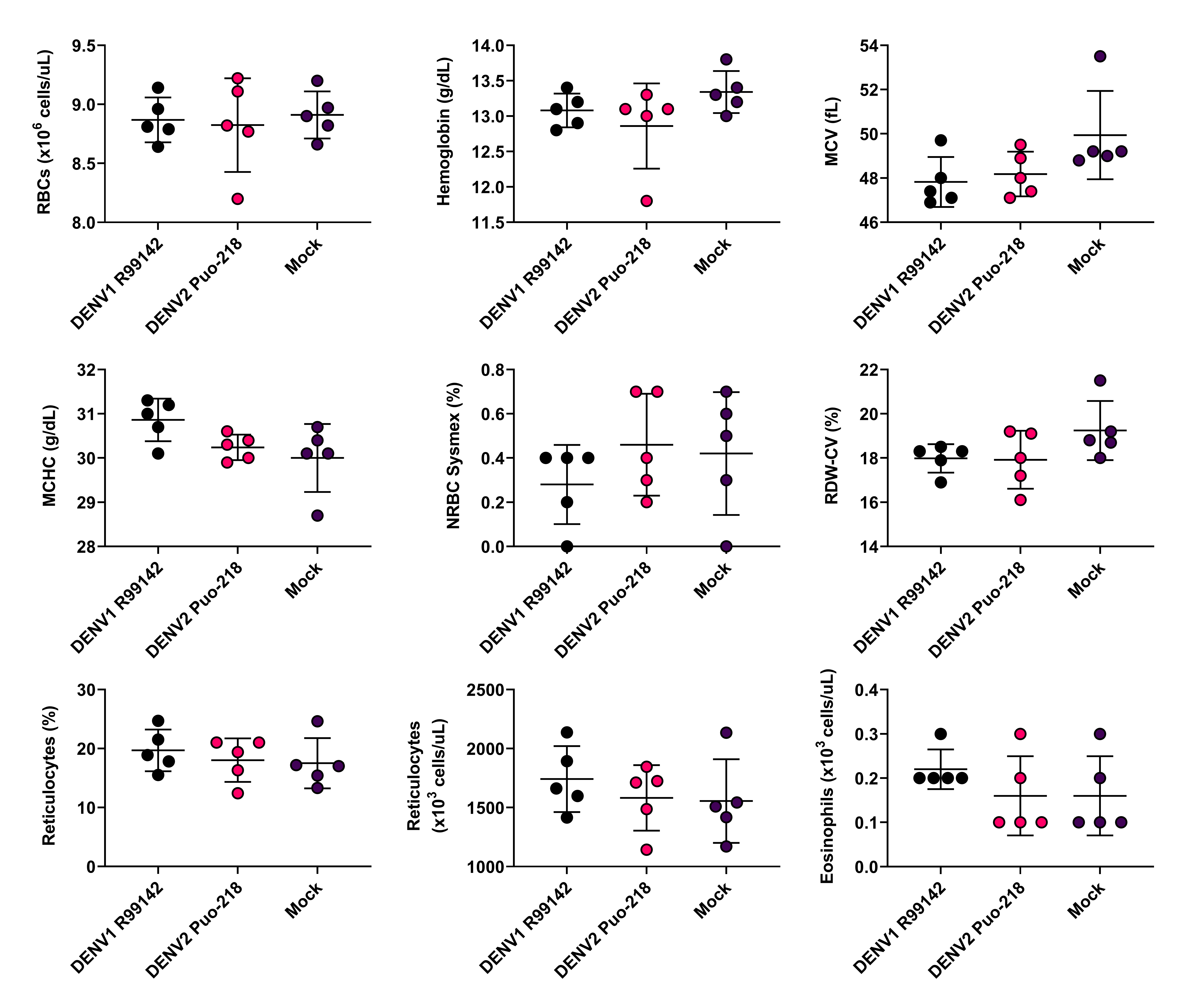

Supplement: Supplementary file 1 — Additional file 1: Figure S1. Hematological changes following dengue virus infection. 10-week old female C57BL/6J mice were treated with 1 mg of antibody to block interferon receptor signaling (IFNAR blocking antibody) and then infected with DENV1 R99142 or DENV2 Puo-218. Hematological analysis was performed 7 days post-infection. Values are means ± SD from groups of 5 animals. Statistical comparisons were made to the mock group using one-way ANOVA with Dunnett’s comparison. Studies were performed in one biological replicate. [file 12915_2020_828_MOESM1_ESM.tif]

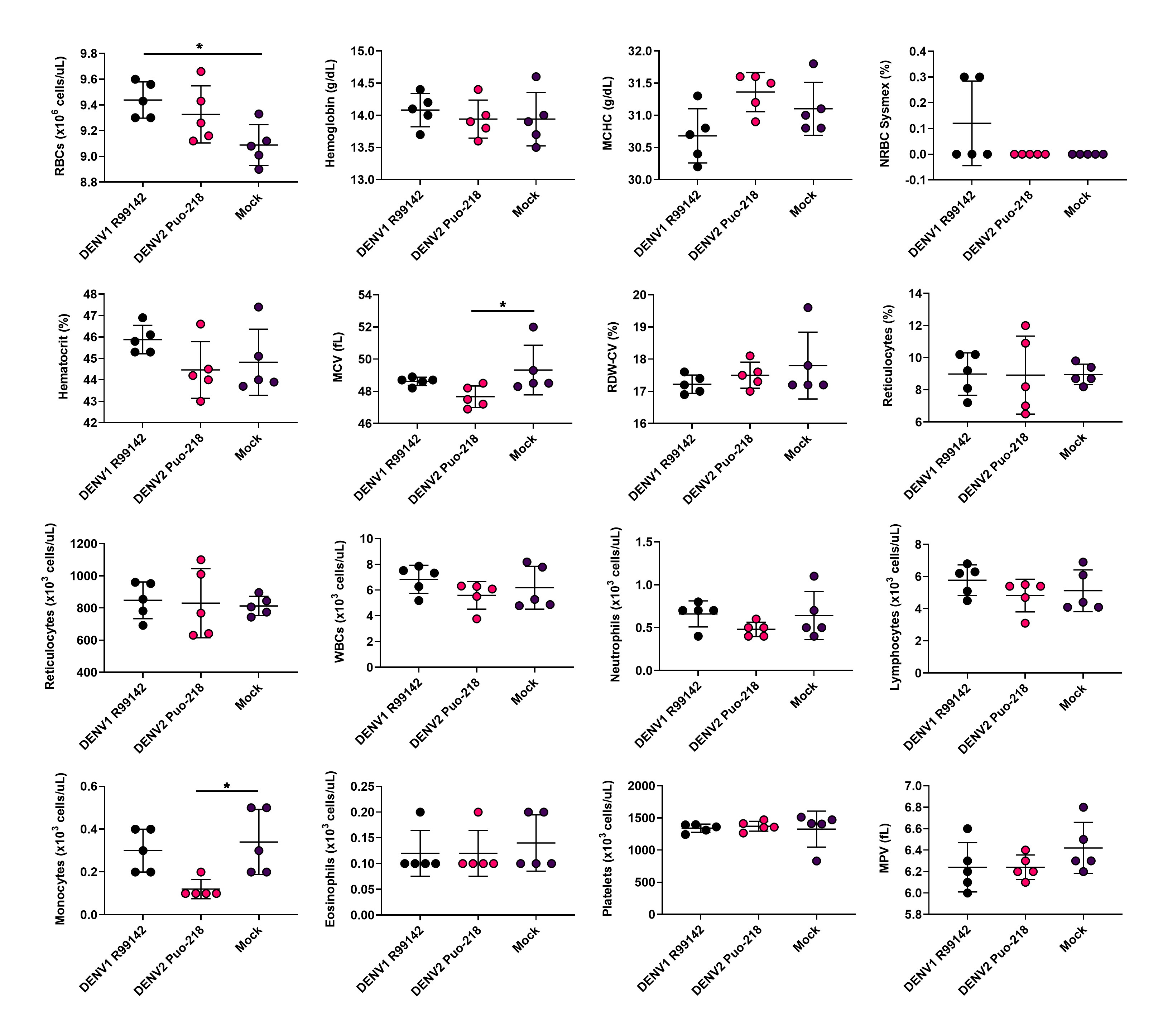

Supplement: Supplementary file 2 — Additional file 2: Figure S2. Hematological changes following dengue virus infection. 10-week old female C57BL/6J mice were treated with 1 mg of antibody to block interferon receptor signaling (IFNAR blocking antibody) and then infected with DENV1 R99142 or DENV2 Puo-218. Hematological analysis was performed 13 days post-infection. Values are means ± SD from groups of 5 animals. Statistical comparisons were made to the mock group using one way ANOVA with Dunnett’s comparison. * indicates p<0.05. Studies were performed in one biological replicate. [file 12915_2020_828_MOESM2_ESM.tif]

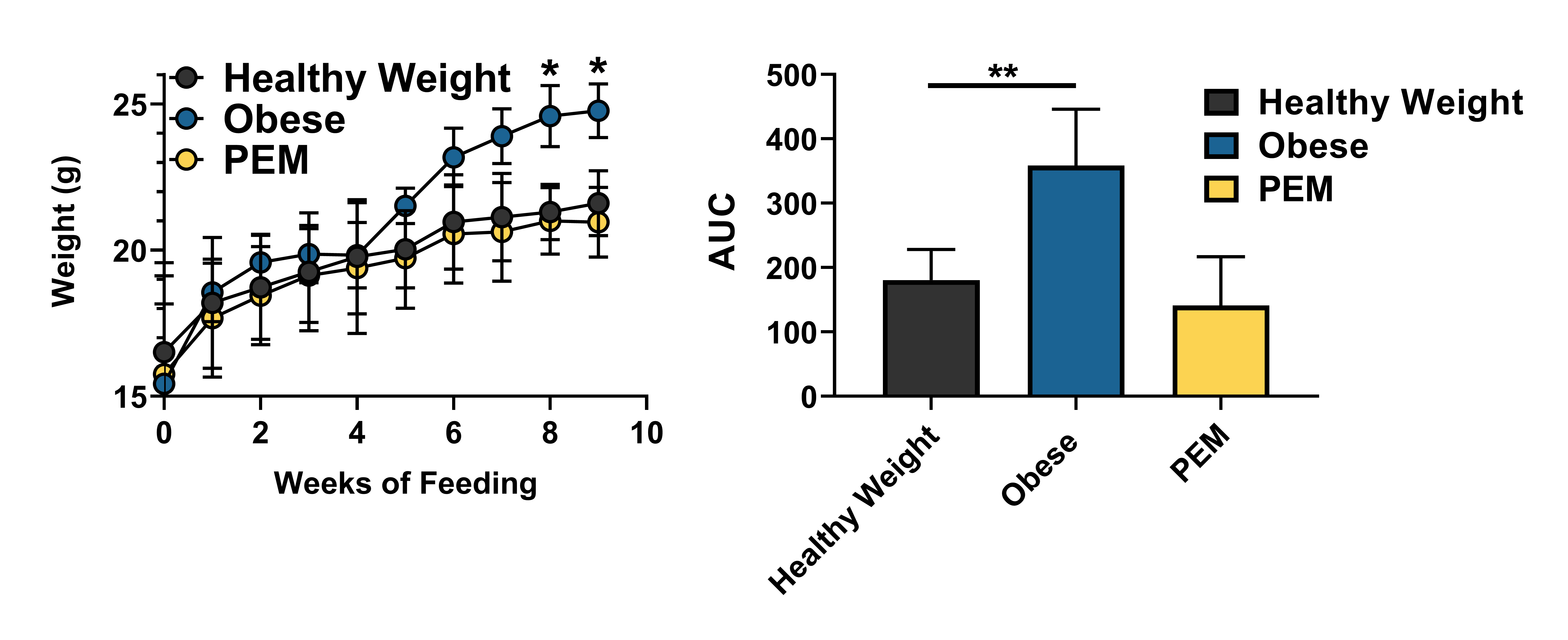

Supplement: Supplementary file 3 — Additional file 3: Figure S3. Weight during feeding before infection. 6-week old female C57BL/6J mice were fed for 8-10 weeks on a control (healthy weight), high-fat (obese), or low-protein (protein-energy malnutrition, PEM) diet. The mice were weighed weekly during feeding. Values are means ± SD from groups of 12-19 animals combined from three independent studies. For studies with a time component, statistical comparisons were made with a two way ANOVA while AUC comparisons were made using a one-way ANOVA, both used Dunnett’s multiple comparisons test to the healthy weight group. * indicates p<0.05, ** indicates p<0.01. [file 12915_2020_828_MOESM3_ESM.tif]

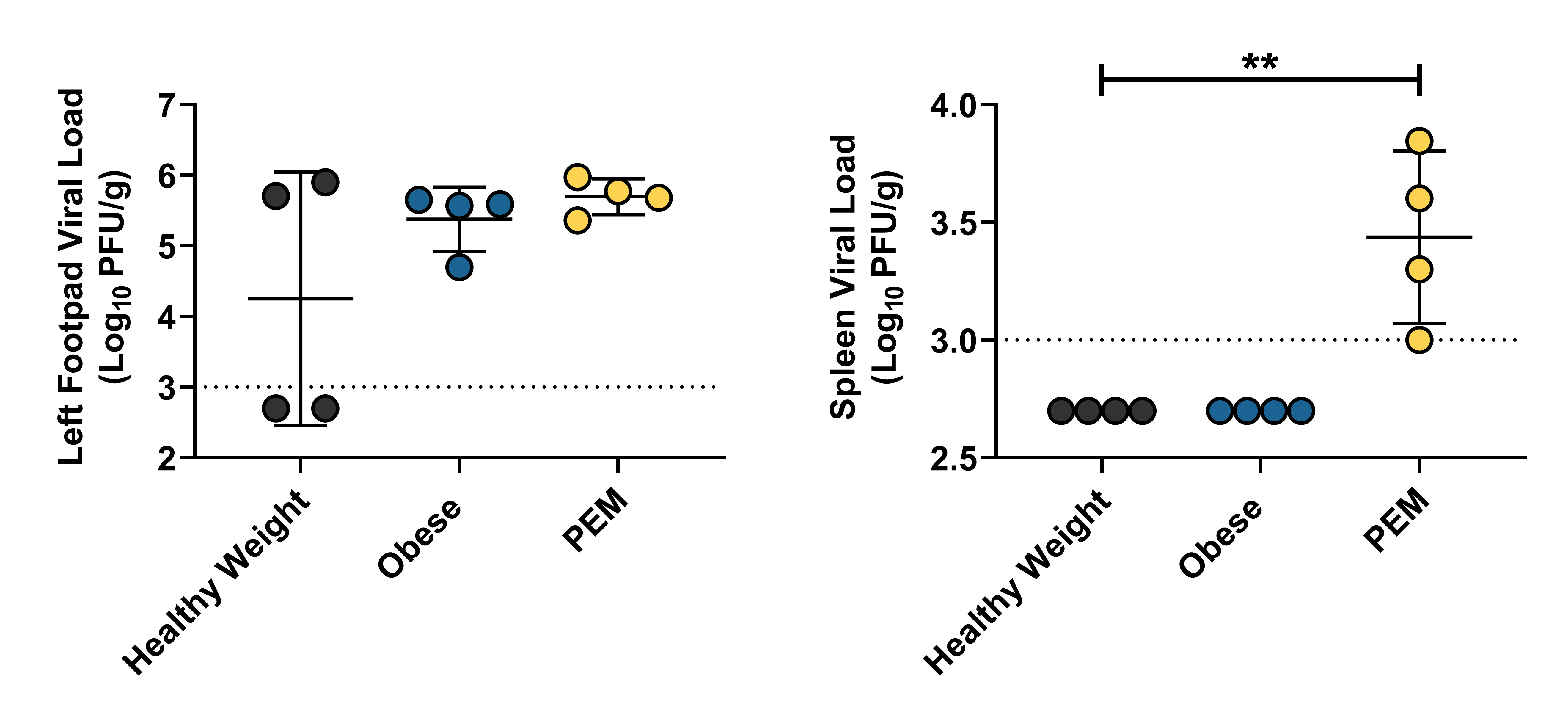

Supplement: Supplementary file 4 — Additional file 4: Figure S4. Organ titers in mice with different nutritional status following dengue virus (DENV) infection. 6-week old female C57BL/6J mice were fed for 8-10 weeks on a control (healthy weight), high-fat (obese), or low-protein (protein-energy malnutrition, PEM) diet and then treated with 1 mg of antibody to block interferon receptor signaling (IFNAR blocking antibody). The next day, mice were infected with DENV2 Puo-218, and three days later, the mice were euthanized, and tissues were collected. Tissue viral load was determined by plaque assay in Vero cells. Values are means ± SD from groups of 4 animals. Statistical comparisons were made to the mock group using one-way ANOVA with Dunnett’s comparison. Studies were performed in one biological replicate. ** indicates p<0.01. The dotted line represents the limit of detection (LOD); all negative samples were given a value of 0.5 x LOD for statistical purposes. [file 12915_2020_828_MOESM4_ESM.tif]

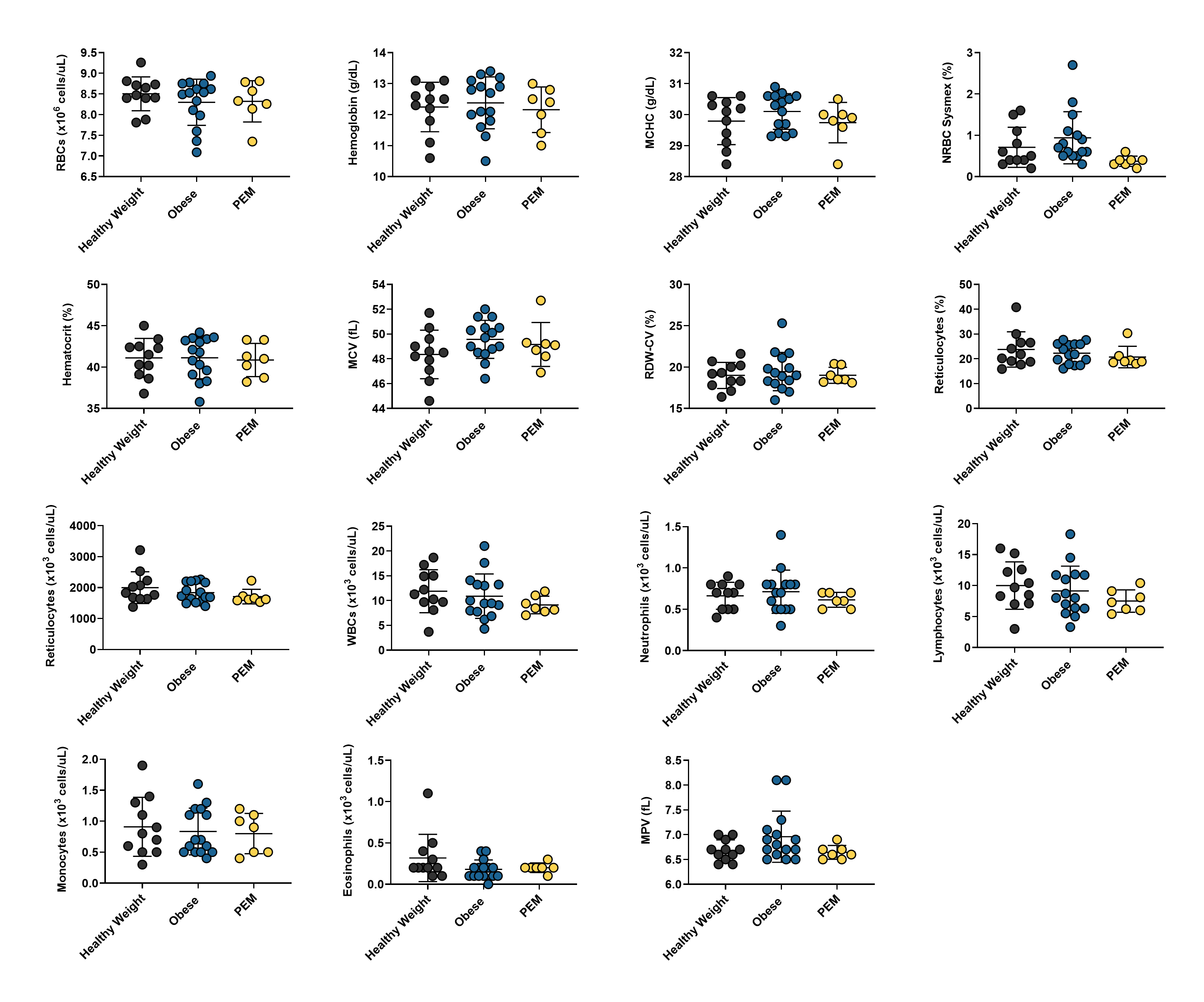

Supplement: Supplementary file 5 — Additional file 5: Figure S5. Hematological changes following dengue virus infection in mice with different nutritional status. 6-week old female C57BL/6J mice were fed for 8-10 weeks on a control (healthy weight), high-fat (obese), or low-protein (protein-energy malnutrition, PEM) diet and then treated with 1 mg of antibody to block interferon receptor signaling (IFNAR blocking antibody). The next day, mice were infected with DENV2 Puo-218, and hematological analysis was performed 7 days later. Values are means ± SD from groups of 7-15 animals. Statistical comparisons were made to the mock group using one-way ANOVA with Dunnett’s comparison. Studies were performed in two biological replicates. [file 12915_2020_828_MOESM5_ESM.tif]

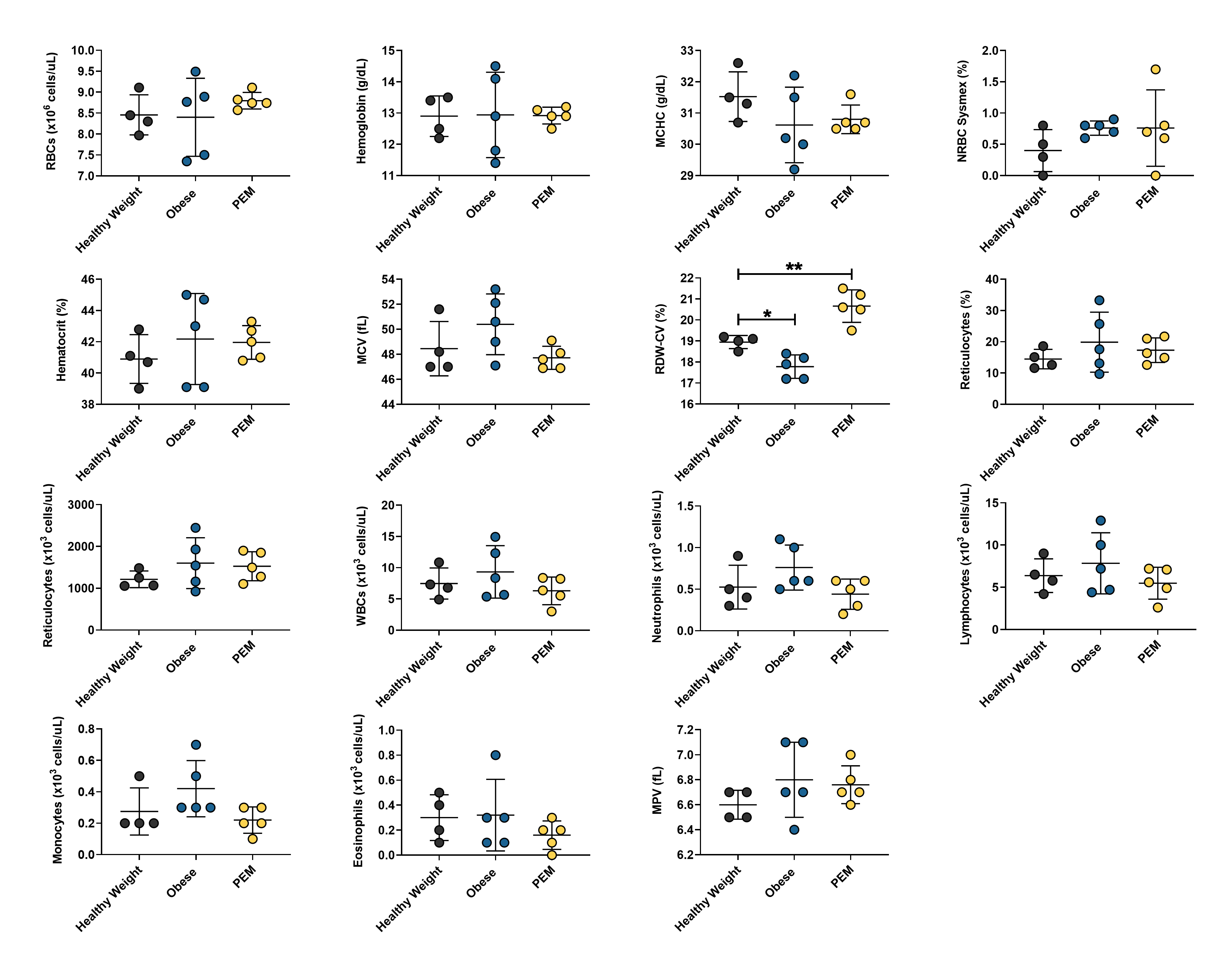

Supplement: Supplementary file 7 — Additional file 7: Figure S6. Hematological changes following secondary dengue virus infection in mice with different nutritional status. 6-week old female C57BL/6J mice were fed for 8-10 weeks on a control (healthy weight), high-fat (obese), or low-protein (protein-energy malnutrition, PEM) diet and then treated with 1 mg of antibody to block interferon receptor signaling (IFNAR blocking antibody). The next day, mice were infected with DENV2 Puo-218. Fiftyfive days later, mice were again treated with 1 mg of IFNAR blocking antibody and then infected with DENV1 R99142. Hematological analysis was performed 7 days post-infection. Values are means ± SD from groups of 4-5 animals. Statistical comparisons were made to the mock group using one-way ANOVA with Dunnett’s comparison. Studies were performed in one biological replicate. * indicates p<0.05. ** indicates p<0.01. [file 12915_2020_828_MOESM7_ESM.tif]
